# Traditional Prostate Cancer Risk Assessment Scales Do Not Predict Outcomes from Brain Metastases: A Population-Based Predictive Nomogram

**DOI:** 10.3390/cancers16173029

**Published:** 2024-08-30

**Authors:** Liliana R. Ladner, Srijan Adhikari, Abhishek S. Bhutada, Joshua A. Cuoco, Vaibhav M. Patel, John J. Entwistle, Cara M. Rogers, Eric A. Marvin

**Affiliations:** 1 Virginia Tech Carilion School of Medicine, 2 Riverside Circle, Roanoke, VA 24016, USA; 2 Department of Neurosurgery, Carilion Clinic, 1906 Belleview Avenue, Roanoke, VA 24014, USA; 3 School of Neuroscience, Virginia Polytechnic Institute and State University, 210 Drillfield Drive, Blacksburg, VA 24061, USA

**Keywords:** brain metastases, nomogram, prostate cancer, prognosis

## Abstract

**Simple Summary:**

Brain metastases from systemic cancer are the most common tumors of the central nervous system. For prostate metastases to the brain, the clinical progression is poorly understood. This retrospective study aims to elucidate clinical risk factors associated with overall survival (OS; months post-diagnosis) in prostate metastases to the brain, and then develop a nomogram to aid in clinical decision making for this vulnerable population. We identified several factors associated with survival, including race, tumor size, and the presence of additional metastases. This study should serve as a clinical framework for prognostication in metastatic prostate cancer to the brain.

**Abstract:**

Brain metastases are an uncommon yet life-limiting manifestation of prostate cancer. However, there is limited insight into the natural progression, therapeutics, and patient outcomes for prostate cancer once metastasized to the brain. This is a retrospective study of 461 patients with metastatic prostate cancer to the brain with a primary outcome of median overall survival (OS). The Surveillance, Epidemiology, and End Results (SEER) database was examined using Cox regression univariate and multivariable analyses, and a corresponding nomogram was developed. The median overall survival was 15 months. In the multivariable analysis, Hispanic patients had significantly increased OS (median OS 17 months, *p* = 0.005). Patients with tumor sizes greater than three centimeters exhibited significantly reduced OS (median OS 19 months, *p* = 0.014). Patients with additional metastases to the liver exhibited significantly reduced OS (median OS 3.5 months, *p* < 0.001). Increased survival was demonstrated in patients treated with chemotherapy or systemic treatment (median OS 19 months, *p* = 0.039), in addition to radiation and chemotherapy (median OS 25 months, *p* = 0.002). The nomogram had a C-index of 0.641. For patients with prostate metastases to the brain, median OS is influenced by race, tumor size, presence of additional metastases, and treatment. The lack of an association between traditional prostate cancer prognosis metrics, including Gleason and ISUP grading, and mortality highlights the need for individualized, metastasis-specific prognosis metrics. This prognostic nomogram for prostate metastases to the brain can be used to guide the management of affected patients.

## 1. Introduction

Brain metastases are the most common central nervous system (CNS) tumors and are associated with high rates of mortality [1,2,3]. Although previously considered unresectable, intracranial metastases have been more frequently treated with surgical resection in recent years, particularly when solitary and mildly symptomatic [4]. However, evidence describing optimal treatments for patients with rare metastases is limited due to small sample sizes. One such rare tumor is metastatic prostate cancer. With few CNS metastases (0.89–3.57%), limited consensus exists on the preferred treatment for patients with metastatic prostate cancer [5,6]. With an aging population, the incidence of prostate metastases to the CNS is increasing, necessitating the standardization of risk assessment and treatment for these patients [7,8].

Prostate cancer is the most common cancer in men, yet brain metastases are rare. Prostate cancer accounted for 1.6 million new diagnoses and 366,000 deaths in 2015 [9]. Risk factors for prostate cancer include family history, elevated androgens, and increasing age [10]. Although prostate-specific antigen (PSA) screening recommendations are evolving, PSA screening globally has increased, resulting in more early-stage diagnoses [9]. The gold standard test for diagnosing prostate cancer is a needle biopsy. Subsequent pathological analysis permits the assignment of a Gleason grade, which is incorporated into risk stratification scales such as the National Comprehensive Cancer Network Risk Stratification or the Pathologic Grading System of the International Society of Urological Pathology (ISUP) [11,12,13]. While beneficial in the prognostication of primary prostate cancer, these scales have limited utility for prostate-derived brain metastases.

Metastatic prostate cancer of the brain is a systemic and debilitating condition, yet few known risk factors exist. Several rare mutations, including splice variants in the androgen receptor (e.g., AR-V7) and known oncogenic mutations (e.g., P53, PTEN, BRCA2), promote treatment resistance in select patients, potentially predisposing them to the development of metastases [14]. Other risk-associated patient demographics include age of presentation around 65 years, concurrent metastases to bone, and classic neurologic symptoms (confusion, headache, memory deficits) [15,16]. Yet, the association of these known clinical features and prostate cancer grading scales with risk of mortality is unknown. Thus, there is a pressing need to elucidate clinical risk factors and optimal treatment modalities to guide clinicians in their assessment of patients with prostate metastases to the brain. 

Large databases facilitate the study of similarly rare diseases. One such database called the SEER database is a cancer surveillance network with demographic, clinical and outcome-related factors for half of the total cancer population in the United States [17]. Utilizing the SEER database, we aimed to identify patients with prostate metastases to the brain, discover outcome-modifying risk factors, and construct a prognostic nomogram for clinical utilization. This clinical tool will support the statistically validated assessment, treatment, and prognostication of patients with prostate metastases to the brain.

## 2. Materials and Methods

### 2.1. Study Design

This study includes patients from the NCI SEER database between 2000 and 2020, from 18 registries across the United States. First, we identified patients with prostate cancer, and then we further stratified those with metastases to the brain. 

### 2.2. Cohort Selection

Clinical characteristics evaluated included the following: age, race, tumor size, PSA value, ISUP grade, additional metastases, timing, type of treatment for the primary prostate tumor, surgical resection of a non-primary site (e.g., lymph node, regional site, or distant site), and surgical resection of brain metastases. Treatments for the primary prostate tumor included combinations of surgery, radiation, and chemotherapy. The age of zero was defined as the patient’s age between birth and one day before age one. The primary outcome was overall survival (OS) or the time between diagnosis and time of death.

### 2.3. Statistical Analyses

Significance was set at *p* < 0.05. In a subset analysis, patients were stratified into two groups based on presence or absence of surgical resection of their brain metastases. To assess for differences between resection groups, descriptive statistics were conducted, including chi-square for categorical variables and Student’s t for numerical variables. For the main analysis, a Cox proportional hazard analysis with the Breslow method for ties was utilized. Univariate analyses were performed on each category to identify clinical characteristics with a statistically significant impact on OS. All significant factors were included in a multivariable proportional hazard model. The unknown tumor size variable was excluded from discussion due to limited clinical relevance. The Kaplan-Meier method was utilized to visualize a subset of survival curves corresponding to the categories that showed significant differences in OS based on the log-rank test. A nomogram was built to assess one- and two-year survival probability based on the results of the Cox multivariable proportional hazard model. The accuracy of the nomogram was evaluated with a C-index, a calibration curve, and an ROC curve. All statistical analyses were performed on R Studio (version 4.2.2) using the “survival”, “survminer”, and “rms” packages.

## 3. Results

### 3.1. Descriptive Data

**Figure 1:**
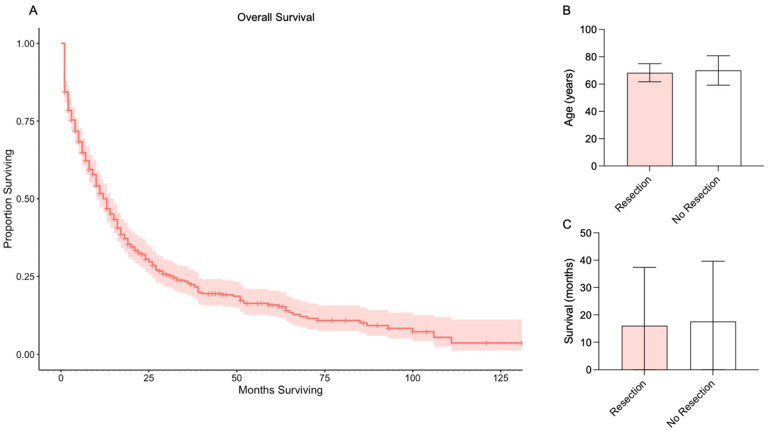
Overall survival and survival by resection. (**A**) Kaplan-Meier survival analysis for all patients with prostate metastases to the brain. Red line indicates median overall survival and red shaded area indicates 95% confidence interval. (**B**) Age of patients with versus without surgical resection of a prostate metastasis to the brain. (**C**) Survival of patients with versus without surgical resection of a prostate metastasis to the brain.

A total of 461 patients were included. Patient characteristics are presented in Table 1. Overall median survival was 13 months (Figure 1A). Most patients were older than 65 years of age (66.16%). Most patients were Non-Hispanic White (63.99%), Non-Hispanic Black (16.49%), Hispanic (13.67%), and Non-Hispanic Asian or Pacific Islander (5.21%). 

Descriptive statistics comparing patients with versus without surgical resection of a brain metastasis from prostate cancer are provided in Table 2. The mean age for patients with surgical resection was 68.33 ± 6.65, compared to 70.02 ± 10.78 for patients without surgical resection (*p* = 0.3175; Figure 1B). Of patients with surgical resection, most were Non-Hispanic White (83%), and only four had additional metastases (two Liver, 11%; two Lung, 11%). Mean overall survival for patients with surgical resection was 16.00 ± 21.36 months and for patients without surgical resection was 17.58 ± 22.04 months (*p* = 0.7621; Figure 1C). 

Tumor size was unreported for most tumors (90.44%), followed by tumors greater than three cm (6.37%). Most patients had PSA values greater than 97.9 ng/mL (57.20%). Most primary prostate tumors were classified as grade five in the ISUP grading scale (55.10%). Additional metastases beyond the brain were most commonly to the bone (56.62%).

The effect of primary prostate tumor treatment characteristics on median OS was subsequently evaluated. Most patients had less than one month from diagnosis to treatment (57.68%). Most patients received no treatment at all (46.64%). Of the remaining treated patients, most had radiation alone (30.59%). Of note, surgical resection in the former analyses included resection of the primary site only (prostate). Accordingly, a subsequent analysis was conducted to determine OS for patients with surgical resection of a metastasis site (e.g., distant site, lymph node). In this analysis, most patients (85.03%) did not undergo resection of a non-primary metastasis site, followed by resection of a distant site such as a metastasis to the bone, liver, lungs, or brain (14.53%). More patients did not undergo surgical resection of a brain metastasis than those who did (96.10% versus 3.90%).

### 3.2. Univariate Analysis

**Figure 2:**
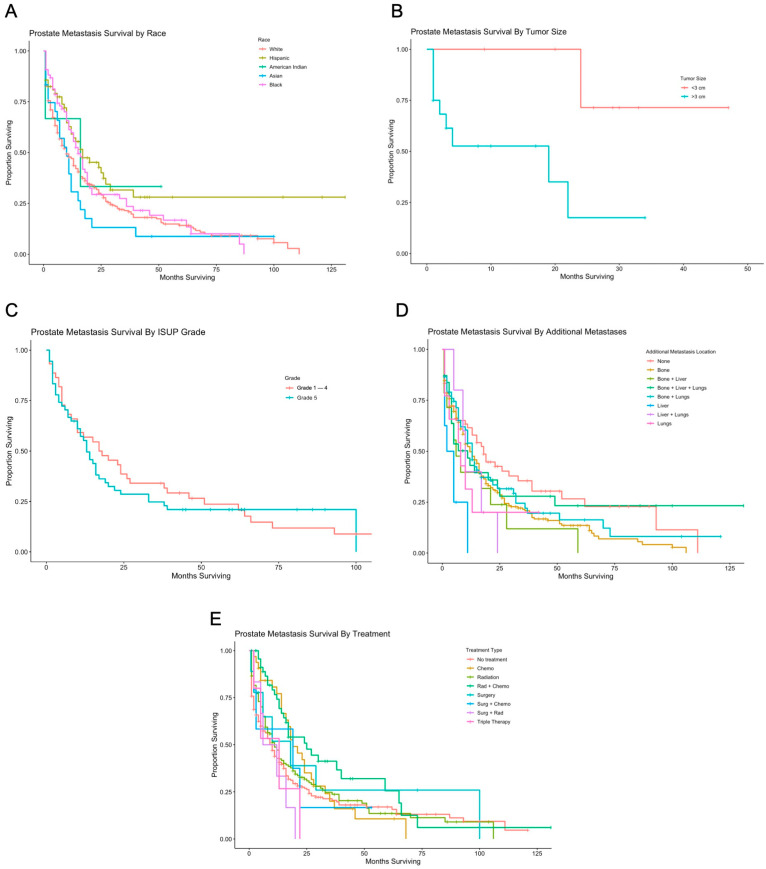
Survival by race, tumor size, ISUP grade, additional metastases, and treatment. (**A**) Kaplan-Meier survival analysis for prostate metastases to the brain, based on race and ethnicity. Median survival for patients who were Non-Hispanic White, Hispanic, Non-Hispanic American Indian/Alaska Native, Non-Hispanic Asian or Pacific Islander, and Non-Hispanic Black were 10, 17, 16, 10, and 16, respectively. (**B**) Kaplan-Meier survival analysis for prostate metastases to the brain based on tumor size. Patients with tumors >3 cm had a median survival of 19 months. (**C**) Kaplan-Meier survival analysis for prostate metastases to the brain based on ISUP grade. Patients with ISUP grade of 1–4 and 5 had median survival of 17.5 and 13, respectively. (**D**) Kaplan-Meier survival analysis for prostate metastases to the brain based on non-primary metastatic sites. Median overall survival was 12, 8, 11, 13, 3.5, 10, and 8 months for patients with additional metastases to the bone, bone and liver, bone and liver and lungs, bone and lungs, liver, liver and lungs, and lungs, respectively. (**E**) Kaplan-Meier survival analysis for prostate metastases to the brain based on treatment. Patients had a median overall survival of 10, 19, 11, 25, 18, 19, 9, and 13 respectively for no treatment, chemotherapy or systemic treatment only, radiation only, radiation and chemotherapy, surgery only, surgery and chemotherapy, surgery and radiation, and triple therapy.

In the univariate analysis, there was no statistical significance seen in OS based on age. Hispanic patients demonstrated a reduced risk of mortality with a median survival of 17 months (95% CI 12 to 39 months, *p* = 0.005), as reflected in Figure 2A. Prostate tumor size greater than three centimeters was associated with significantly increased risk of mortality (19 months with 95% CI not yet reached, *p* = 0.019), as seen in Figure 2B. 

There was no statistically significant difference in OS based on PSA value or ISUP grading (Figure 2C). Patients with concurrent metastases to the bone exhibited a significantly increased median OS of 12 months (95% CI 10 to 16 months, *p* = 0.042), and patients with metastases to the liver exhibited a significantly increased median OS of 3.5 months (95% CI not yet reached, *p* = 0.0003), as seen in Figure 2D. Patients treated with radiation and chemotherapy exhibited significantly improved median OS of 25 months (95% CI 16 to 65 months, *p* = 0.002), as seen in Figure 2E. There was no difference in median OS for patients with surgical resection of a distant site, such as bone, liver, lung, or brain metastases, or for patients with resection of brain metastases (Table 1).

### 3.3. Multivariable Analysis

**Figure 3:**
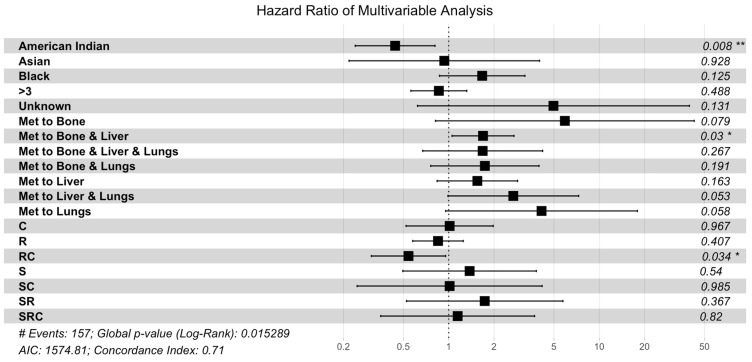
Multivariable Cox proportional hazard analysis. The multivariable analysis included race, tumor size, multi-organ metastases, and treatment. Abbreviations: C, chemotherapy or systemic treatment only; R, radiation only; RC, radiation and chemotherapy; S, surgical resection; SC, surgery and chemotherapy; SR, surgery and radiation; SRC, surgery and radiation and chemotherapy (** represents *p* < 0.01, * represents *p* < 0.05).

A multivariable analysis was performed for all factors significantly influencing OS in the univariate analysis, including patient race, tumor size, and presence of additional metastases (Table 3, Figure 3). In the multivariable analysis, factors that remained significant included Hispanic race (*p* = 0.0052), tumor size greater than three cm (*p* = 0.0147), metastases to the liver (*p* = 0.0004), and treatment with radiation and chemotherapy (*p* = 0.0015). Factors that gained significance in the multivariable analysis included treatment with chemotherapy or systemic therapy (*p* = 0.0385). Significance was lost in the group with additional metastases to the bone (*p* = 0.755).

### 3.4. Nomogram and Validation

**Figure 4:**
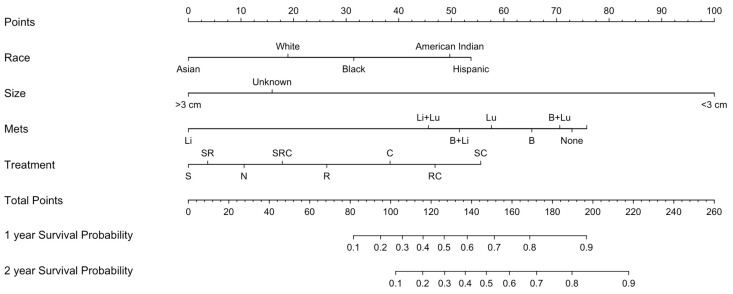
Nomogram for survival in prostate metastases to the brain. The nomogram incorporates statistically validated factors based on a multivariable Cox proportional hazard model (*p* < 0.05). Variables found to be significant include race, tumor size, presence of additional metastases, and treatment. Abbreviations: N, no treatment; C, chemotherapy or systemic treatment only; R, radiation only; RC, radiation and chemotherapy; S, surgical resection; SC, surgery and chemotherapy; SR, surgery and radiation; SRC, surgery and radiation and chemotherapy.

**Figure 5:**
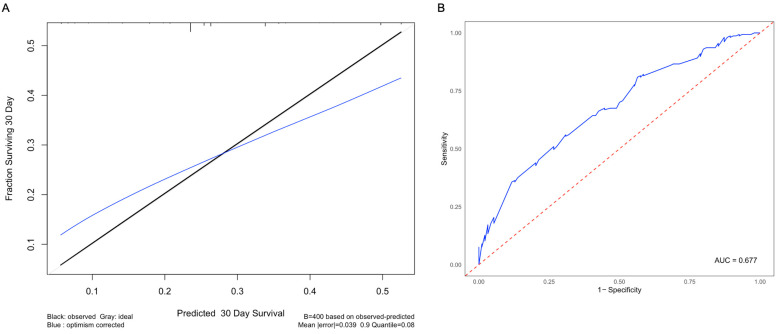
Calibration curve and receiver operating characteristic curve for the nomogram. (**A**) To compare observed survival (in black) with corrected survival (in blue), a calibration curve for 30-day survival was generated. (**B**) The ROC curve was built to evaluate the performance of the nomogram, with an AUC value of 0.677. The blue line represents the ROC curve for this model, and the red line represents the threshold for a random guess.

The nomogram included race, tumor size, and the presence of additional metastases predictors of patient survival (Figure 4). The nomogram had a C-index of 0.641, indicating a good concordance between the predicted and observed outcomes (Figure 5A). The nomogram-based model had good performance with an AUC of 0.677 (Figure 5B).

## 4. Discussion

Prostate metastases to the brain are rare, and factors that influence survival are poorly understood. In this study, we demonstrate that traditional prostate cancer prognosis metrics, such as the ISUP scale, are not indicative of prognosis for intracranial metastases; rather, prognosis is driven by factors such as race, tumor size, and the presence of additional metastasis sites. Moreover, we characterize patients who undergo surgical resection of these metastases. This nomogram-based prognostic model is a novel addition to the literature. 

### 4.1. Traditional Prostate Cancer Risk Predictors

Traditional prostate cancer grading scales lack clinical utility in assessing risk for progression and survival from metastases to the brain. Two well-accepted prostate cancer prognosis models are the Gleason and ISUP scales. The Gleason grade ranges from two to ten, reflects histologic features of a prostate tumor, and can predict progression to metastatic cancer, survival, and treatment response [18]. In 2005, a new scale was developed that better predicts capacity for tumor resection, called the ISUP grade [19]. Despite the clinical utility of these scales in the management of primary prostate cancer, neither were developed to predict outcomes from metastatic prostate cancer. Accordingly, this study failed to show an association of overall survival with Gleason or ISUP grades of the primary prostate tumor. On the contrary, a 2014 study suggested that Gleason scores are the strongest prognostic factor for survival in metastatic prostate cancer [20]. It is possible that the low number of patients in this study with brain metastases reduces the chance of finding a true difference, or that these prostate-specific models do not account for the unique nature of intracranial metastases. Moreover, the utility of Gleason scoring may differ across metastasis sites with varied genetic profiles and asynchronous growth patterns [21]. Traditional prostate cancer prognosis metrics have limited efficacy for patients with brain metastases, but future prospective studies with larger patient sample sizes may prove useful.

### 4.2. Race and Ethnicity

Ethnicity is implicated in the development of primary prostate cancer and subsequent brain metastases. In this study, we demonstrate a reduced risk of mortality in Hispanic patients and an increased risk of mortality in Asian or Pacific Islander patients. In contrast to the notion that African American men have a greater risk of primary prostate cancer diagnosis and mortality, we demonstrate no other race-related differences in mortality [22]. Moreover, outcomes for Hispanic patients vary widely across race and location-related subgroups, as black Hispanic patients have a greater risk of mortality than white Hispanic patients and the incidence of prostate cancer is increasing in Latin American countries [23,24]. In African American patients, limited access contributes to delayed presentation and poor outcomes [25]. Other minority groups, including Asian Americans, Native Hawaiians, and Pacific Islanders, present with extensive disease progression due to delayed diagnosis [26]. This is concordant with our finding of significantly decreased overall survival in Asian or Pacific Islander patients. As there is no race-based difference in patients who undergo surgical resection of a brain metastasis, these disparities can be attributed to multiple tumor-specific and societal factors, including access to care, genetic susceptibility, and cultural preferences [27,28]. Although it is known that race influences the risk of developing primary prostate cancer and prostate metastases, there are few studies describing race-related outcomes in brain-specific metastases [29]. 

### 4.3. Tumor Size

Tumor size influences survival, as patients with larger primary prostate tumors have a greater risk of mortality from brain metastases. Prostate tumor maximum diameter is associated with recurrence after prostatectomy, presence of distant metastases, and mortality after salvage radiation [30,31,32]. Larger tumors are at higher risk of local lymphatic invasion and subsequent systemic seeding [33]. Moreover, the capacity for surgical resection of any brain metastasis inversely correlates with lesion size [34]. Accordingly, the size of the primary prostate tumor and brain metastasis drives treatment response, and an optimal prognosis model should incorporate tumor size. 

### 4.4. Concurrent Metastases

Prostate cancer with multiple metastasis sites reflects aggressive disease. This study shows that, in addition to intracranial metastases, concurrent metastases to the bone or liver are associated with reduced overall survival. Additionally, patients with single metastatic disease to the brain have improved survival, and those who undergo surgical resection of their brain metastases are most likely to have no other metastasis sites. At diagnosis, prostate cancer commonly presents with bone metastases (10%) [35]. The likelihood of systemic tumor seeding is dependent on primary tumor histology, as the proliferation of cells with stem-like properties predisposes to the development of metastatic cancer [36,37]. Additionally, immune mediators in the tumor microenvironment can influence tumor development and metastasis [38]. Due to tumor cell pluripotency and local immune mediators, patients with metastases are at risk for poor outcomes at the time of primary prostate cancer diagnosis [39,40,41]. Further research on the tumor microenvironment and stem-cell-like histologic features is necessary to predict the risk of metastases and mortality in affected patients [42]. Moreover, their quantification at initial diagnosis would permit the development of a histopathologic metastasis prediction model.

### 4.5. Treatment

Various treatment modalities, when appropriately selected, can improve survival in metastatic prostate cancer to the brain. Of note, this study demonstrates that this rare tumor type may be responsive to chemotherapy or other systemic treatments, as well as radiation in combination with chemotherapy, as reflected by increased survival. In localized prostate cancer, surgery and radiotherapy may suffice, but metastatic prostate cancer is managed with androgen deprivation therapy, salvage radiotherapy, and chemotherapy, as corroborated by this study [43]. Moreover, the combination of multiple types of androgen deprivation therapy and chemotherapies may provide synergistic treatment effects [44,45,46,47]. Although there are insufficient patients to perform a national trial investigating the efficacy of specific treatment regimens on survival in metastatic prostate cancer to the brain, this study highlights the promising role of chemotherapy, systemic treatments, and combination therapy. 

### 4.6. Predictive Modeling

Nomograms represent an advancement in prognostic data modeling and are increasingly utilized to enhance prognostication for patients with otherwise rare and understudied conditions. This nomogram incorporates a novel predictive model for prostate metastases to the brain. Future modifications to this nomogram, informed by an expanding sample size, hold promise for improved predictive modeling.

The area under the curve (AUC) of 0.677 suggests that this model appropriately discriminates between patients who are at low risk and high risk of mortality from intracranial prostate metastases. Although a higher AUC is desirable, this is the first model to date to predict survival from intracranial metastases from the prostate, and, as such, establishes a precedent. Future studies that include more patients and account for clinical confounders will improve the performance of the model.

### 4.7. Limitations and Generalizability

Although efforts were made to mitigate limitations to the current study, there are several which remain. Granular patient-specific details are not provided in the SEER database. Also, this study would be improved if it could include specific locations of prostate metastases within the central nervous system, number of metastases, or timing and dosage of each treatment (e.g., surgeries, chemotherapy cycles, non-chemotherapy systemic treatment regimen, radiation types). Additionally, because this study has a limited number of patients and the SEER database only accounts for 47.9% of the United States (US) population, this study has limited generalizability to the broader US or global population. Moreover, the SEER database only includes information on cancer cases and, as such, may not accurately reflect risk for the general population. 

## 5. Conclusions

This is the first study to develop a prognostic nomogram for survival from prostate metastases to the brain. We demonstrate that survival is not influenced by traditional prostate cancer prognosis metrics like ISUP grading; rather, race, tumor size, the presence of additional metastases, and treatment influence prognosis. These findings provide support for a personalized approach to the management of prostate metastases to the brain, regardless of ISUP score.

## Figures and Tables

**Table 1:** Clinical demographics, tumor characteristics, and treatment regimen for patients with prostate metastases to the brain.

Category	Subcategory	Patients	Percentage	Survival (Months)	[95% CI]	Univariate			
						HR	[95% CI]	*p*	Sig
Overall		461	100.00	13	[11–15]				
Age	0–64	156	33.84	14	[11–18]	reference			
	65+	305	66.16	11	[9–14]	1.167	[0.9346–1.457]	0.173	
Race	Non-Hispanic White	295	63.99	10	[8–14]	reference			
	Hispanic (all races)	63	13.67	17	[12–39]	0.6008	[0.4203–0.8588]	0.005	**
	American Indian/Alaska Native	3	0.65	16	[0.5–NA]	0.6942	[0.1724–2.7952]	0.608	
	Asian or Pacific Islander	24	5.21	10	[6–16]	1.2317	[0.7874–1.9269]	0.361	
	Black	76	16.49	16	[13–19]	0.8441	[0.6327–1.1260]	0.249	
Tumor Size (cm)	<3	8	3.19	NA	[NA–NA]	reference			
	>3	16	6.37	19	[2–NA]	11.699	[1.478–92.58]	0.020	*
PSA value (ng/mL)	<97.9 ng/mL	104	42.80	11	[7–16]	reference			
	>97.9 ng/mL	139	57.20	14	[11–19]	0.9219	[0.7005–1.213]	0.561	
ISUP Grading (TURP)	Grade 1–4	44	44.90	17.5	[10–19]	reference			
	Grade 5	54	55.10	13.0	[10–38]	1.118	[0.720–1.735]	0.619	
Additional Metastases	None	66	14.32	18	[13–39]	reference			
	Bone	261	56.62	12	[10–16]	1.40859	[1.0119–1.961]	0.042	*
	Bone and Liver	14	3.04	8	[4–NA]	1.70839	[0.8794–3.319]	0.114	
	Bone and Liver, and Lungs	31	6.72	11	[5–49]	1.023939	[0.6099–1.737]	0.904	
	Bone and Lungs	67	14.53	13	[11–22]	1.21943	[0.8107–1.834]	0.341	
	Liver	8	1.74	3.5	[1–NA]	4.39485	[1.9580–9.864]	0.001	***
	Liver and Lungs	5	1.08	10	[9–NA]	1.90014	[0.7503–4.812]	0.176	
	Lungs	9	1.95	8	[3–NA]	1.75303	[0.7868–3.906]	0.170	
Time to Treatment	<1 month	214	57.68	14	[11–18]	reference			
	1–18 month	157	42.32	14	[12–17]	0.8217	[0.6461–1.045]	0.109	
Treatment Summary	No treatment	215	1.95	10	[8–13]	reference			
	Chemotherapy or Systemic Treatment	32	6.94	19	[16–33]	0.7407	[0.4890–1.1220]	0.157	
	Radiation only	141	30.59	11	[8–15]	0.8889	[0.6974–1.1330]	0.341	
	Radiation and Chemotherapy	48	10.41	25	[16–65]	0.5427	[0.3654–0.8061]	0.002	**
	Surgery only	9		18	[6–NA]	0.7144	[0.3344–1.5261]	0.385	
	Surgery and Chemotherapy	5	1.08	19	[3–NA]	0.8062	[0.2987–2.1760]	0.6707	
	Surgery and Radiation	6	1.30	9	[5–NA]	1.5082	[0.6663–3.4139]	0.3242	
	All Three	5	1.08	13	[5–NA]	1.3411	[0.4965–3.6225]	0.5626	
Surgery to Non-Primary Site	No/unknown/other	392	85.03	12	[10–14]	reference			
	Distant Site	67	14.53	21	[10–33]	0.7486	[0.5528–1.014]	0.061	
	Lymph Node	2	0.43	0.5	[0.5–NA]	2.0611	[0.2884–14.732]	0.471	
Surgery to Brain Metastasis	No	443	96.10	13	[11–15]	reference			
	Yes	18	3.90	7.5	[2–NA]	1.1459	[0.671–1.957]	0.618	

Univariate Cox proportional hazard analyses for each prognostic factor (*** represents *p* < 0.001, ** represents *p* < 0.01, * represents *p* < 0.05). NA indicates the limit is not yet reached.

**Table 2:** Comparison of clinical demographics and tumor characteristics for patients with versus without surgical resection of a prostate metastasis to the brain.

Category	Subcategory	Resection (*n* = 18)	No Resection (*n* = 443)	*p*	Sig
Age		68.33 ± 6.65	70.02 ± 10.78	0.3175	
Race				0.3914	
	White	15 (83%)	280 (63%)		
	Hispanic	0 (0%)	63 (14%)		
	American Indian/Alaska Native	0 (0%)	3 (0.6%)		
	Asian or Pacific Islander	1 (6%)	23 (5%)		
	Black	2 (11%)	74 (17%)		
Additional Metastases				<0.001	***
	None	14 (78%)	51 (12%)		
	Bone	0 (0%)	261 (59%)		
	Bone + Liver	0 (0%)	14 (3%)		
	Bone + Liver + Lungs	0 (0%)	31 (7%)		
	Bone + Lungs	0 (0%)	67 (15%)		
	Liver	2 (11%)	7 (2%)		
	Liver + Lungs	0 (0%)	4 (1%)		
	Lung	2 (11%)	8 (2%)		
Survival		16.00 ± 21.36	17.58 ± 22.04	0.7621	

Student’s t and chi-square tests for each prognostic factor (*** represents *p* < 0.001).

**Table 3:** Multivariable analysis with clinical demographics, tumor characteristics, and treatment regimen for patients with prostate metastases to the brain.

Category	Subcategory	Multivariable			
		HR	[95% CI]	*p*	Sig
Race	Non-Hispanic White	reference			
	Hispanic (all races)	0.4126	[0.2216–0.7680]	0.0052	**
	Non-Hispanic American Indian/Alaska Native	0.4956	[0.1172–2.0960]	0.3400	
	Non-Hispanic Asian or Pacific Islander	1.7181	[0.8707–3.3904]	0.1186	
	Non-Hispanic Black	0.7364	[0.4721–1.1485]	0.1772	
Tumor Size (cm)	<3	reference			
	>3	13.5045	[1.6672–109.3849]	0.0147	*
Additional Metastases	None	reference			
	Bone	1.2316	[0.7508–2.0202]	0.4094	
	Bone and Liver	1.7555	[0.6909–4.4609]	0.2369	
	Bone and Liver and Lungs	1.0037	[0.4322–2.3311]	0.9931	
	Bone and Lungs	1.0873	[0.5749–2.0564]	0.7968	
	Liver	6.3700	[2.2858–17.7522]	0.0004	***
	Liver and Lungs	2.2564	[0.5131–9.9225]	0.2815	
	Lungs	1.5616	[0.5998–4.0660]	0.3613	
Treatment Summary	No treatment	reference			
	Chemotherapy or Systemic Treatment Only	0.4883	[0.2477–0.9627]	0.0385	*
	Radiation Only	0.6775	[0.4579–1.0024]	0.0514	
	Radiation and Chemotherapy	0.3946	[0.2220–0.7014]	0.0015	**
	Surgery Only	1.2845	[0.4453–3.7057]	0.6432	
	Surgery and Chemotherapy	0.3267	[0.0776–1.3751]	0.1271	
	Surgery and Radiation	1.2557	[0.3678–4.2866]	0.7163	
	All Three	0.8085	[0.2501–2.6143]	0.7226	

Multivariable Cox proportional hazard analyses for each prognostic factor (*** represents *p* < 0.001, ** represents *p* < 0.01, * represents *p* < 0.05).

## Data Availability

The data presented in this study are openly available in the SEER database at https://seer.cancer.gov, accessed on 10 July of 2023.
